# Knowledge, Attitude, and Practice Toward COVID-19 Among Sherubtse College Students in Bhutan: A Web-Based Cross-Sectional Study

**DOI:** 10.3389/fpubh.2021.721493

**Published:** 2021-11-17

**Authors:** Thinley Dorji, Karma Wangmo, Yezer, Tashi Wangchuk, Tshokey, Kinley Wangdi

**Affiliations:** ^1^Kanglung Hospital, Trashigang, Bhutan; ^2^Regional Livestock Development Centre, Kanglung, Bhutan; ^3^Sherubtse College, Royal University of Bhutan, Trashigang, Bhutan; ^4^Mongar Higher Secondary School, Mongar, Bhutan; ^5^Jigme Dorji Wangchuck National Referral Hospital, Thimphu, Bhutan; ^6^Department of Global Health, Research School of Population Health, College of Health and Medicine, Australian National University, Canberra, ACT, Australia

**Keywords:** knowledge, attitude, practice, COVID-19, Bhutan, coronavirus

## Abstract

Bhutan has reopened schools and colleges after an initial closure to contain coronavirus disease 2019 (COVID-19) transmission. However, the risk of transmissions is higher in the schools and colleges due to crowding. Therefore, this study aimed to assess the level of knowledge, attitude, and practice (KAP) toward COVID-19 among the students of Sherubtse College in Bhutan. A cross-sectional study using a questionnaire was conducted in September 2020 among the students of Sherubtse College, Bhutan. The questionnaire was made in the Google Forms and administered through a social forum WeChat app. The KAP scores were calculated that include mean scores. The association between the KAP was assessed using the Pearson's correlation coefficient. A total of 613 students participated in the survey. The majority of the participants (57%) were female and 56% were from the third year. The mean knowledge score was 10.7 (SD = 1.7; range 0–14), mean attitude score of 3.67 (SD = 1.0; range: 0–5), and mean practice score of 5.19 (SD = range: 0–6). A majority of the students had good knowledge (98%) and practice (93.5%) scores, and a positive attitude (86.6%) toward COVID-19. A positive but weak correlation between good knowledge and practice (*r* = 0.1, *p* = 0.0126) was observed. Having a positive attitude led to practicing appropriately most of the time (*r* = 0.1866, *p* < 0.001). The students had good KAP scores and followed the COVID-19 prevention protocols advocated by the government. Good knowledge and a positive attitude were translated into good practice. Therefore, the education campaign of the Bhutan government seems to be effective in the students.

## Introduction

Coronavirus disease 2019 (COVID-19) pandemic, caused by the new coronavirus strain severe acute respiratory syndrome coronavirus 2 (SARS-CoV-2), has infected more than 172 million people as of June 7, 2021 (1) with 2.93 million deaths. As a result, it is one of the serious public health problems worldwide in the Twenty first century (1, 2). The first case of COVID-19 in Bhutan was reported on March 5, 2020 (3, 4) and the current record stands (7 June 2021) at 1,687 cases with one death (5). As in other countries around the world, the COVID-19 pandemic has brought communities to a standstill due to the lockdowns to prevent outbreaks.

During the initial phase of the pandemic, each country adopted various responses to COVID-19 to slow transmission and to prevent oversaturation of the healthcare systems (6–9). Bhutan adopted proactive prevention methods to limit community transmission. This included a mandatory facility quarantine for returning traveler for 2 weeks from March 16, 2020 (10). It was later extended to 3 weeks upon the recommendation from the National COVID Task Force. In addition, the international borders were sealed from March 23, 2020 except for the supply of essential goods (3, 4), which was done under the strict COVID-19 prevention protocols, such as disinfection of vehicles and strict social distancing between delivery and trans-shipping personals. Further, the government has undertaken various preventive measures to stop the spread of the disease, such as hand washing, social distancing, and the use of face mask in public places (11). The public is encouraged to use the “Druk Trace” app, for contact tracing. Regular COVID-19 screenings were instituted among the frontline workers (police, foresters, and volunteers), students, and business community. All the patients undergo mandatory COVID-19 testing before admissions to the hospitals. The flu clinics for screening COVID-19 are set up to prevent the patients from visiting the hospitals. Moreover, the government has instituted enhanced COVID-19 surveillance whereby 10% of health workers, frontline workers, and school students need to be tested for COVID-19 fortnightly (12).

The schools and colleges have reopened after the initial lockdown with self-containment in place. The students are required to adhere to the normal prevention and control measures, such as social distancing, regular washing of hands and isolation, and testing for COVID-19 for any suspected signs and symptoms. Despite these safeguards against COVID-19, the colleges and schools are at risks for outbreaks due to the congregation of many people. This is evident by recent COVID 19 outbreak school (13) and college (14) in Bhutan. Therefore, knowledge, attitude, and practice (KAP) in the colleges and schools should be at the optimum in averting the outbreaks. The success of preventive measures initiated by the government depends on the uptake and adherence to these preventive measures (15, 16), which is determined by the level of knowledge toward the disease (17, 18). The prior KAP studies on COVID-19 among the university students in other countries were variable (19–21). However, no such studies were done in Bhutan and this study aimed to assess the KAP on COVID-19 among the students of Sherubtse Colleges in Bhutan to inform the policymakers for making informed decisions in the future.

## Methods

### Study Design and Setting

**Figure 1:**
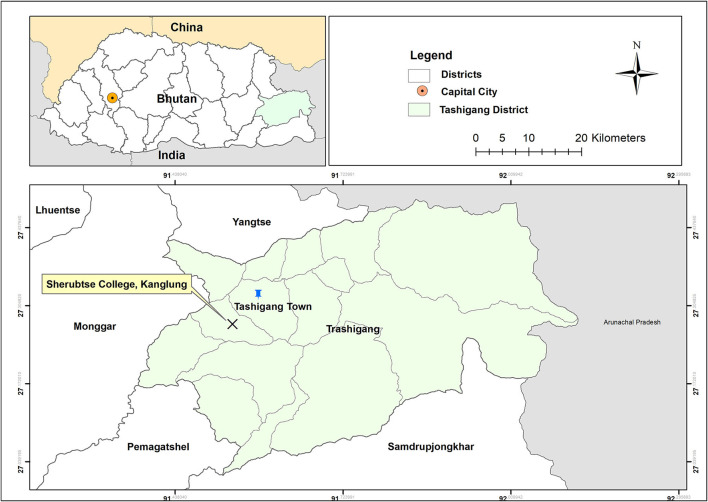
Map of Sherubtse College, Kanglung, Trashigang District, Bhutan.

A cross-sectional study was conducted among the students of Sherubtse College in September 2020 using a web-based survey. A face-to-face survey was not feasible due to the social distance imposed by the Royal Government of Bhutan. Sherubtse College is the oldest college in Bhutan, located in Kanglung under Trashigang District in eastern Bhutan (Figure 1). For the 2020 academic year, there were 1,574 students of whom 52% were women. College offers five courses on Science, Mathematics, and Arts. Majority of the students reside in the college hostels and are allowed limited interaction with the general public.

### Sampling and Participant Recruitment

We calculated the sample size using the following formula:


$$\begin{array}{l} N=\;\frac{z^{2}pq}{d^{2}} \end{array}$$

Where,

*N* is the number of study participants;

*z* is the value of the statistic in a normal distribution for a 95% *CI* (this value was 1.96 for this study);

*p* is the prevalence estimate set at 0.5;

*q* = (1–p);

*d* is the precision of the prevalence estimate set at 0.05.

The required sample size was 384. However, we invited all the students to participate in this study.

The survey questionnaire was developed in the Google survey tool (Google Forms). The link generated from the Google Form was circulated to the students through social media groups via WeChat app. In addition, the link was shared with the students through the contact list of the researchers. The link led to the first page of the Google Form, which contained a summary of the survey, such as research background, aims, and expected outcomes. At the end of the first page, there was a declaration of confidentiality and an informed consent of the participants if they voluntarily agree to participate in the study.

### Questionnaire and Scoring

The data was collected using a pretested questionnaire adapted from a similar study in China by Zhong et al. (18). Moreover, the questionnaire was pre-tested among the 20 college students, and a questionnaire was modified accordingly. This group of students was asked not to participate in the main study. The questionnaire was divided into four parts: (i) socio-demographic characteristic; (ii) knowledge domain; (iii) attitude domain; and (iv) practice section. The demographic questions included age, sex, year of study, and the department. There were 14 knowledge questions, such as four on clinical presentations, three on transmission routes, and four on the prevention and control of COVID-19. Five attitude questions were on insecurity, optimism, confidence, and responsibility. The six practice questions were on the use of the COVID-19 tracing app (Druk Trace), appropriate personal hygiene, such as hand washing, wearing a face mask, and avoiding going to public places.

For knowledge, the participants received one point for each correct answer and zero for an incorrect answer (range 0–14). Knowledge score of ≤7 (50%) and >7 was classified as poor and good knowledge, respectively. For practice, every correct answer was awarded one point while for a negative response was awarded zero points (range 0–6). A minimum of 3 (50%) of practice score was considered good practice. The attitude questions consisted of two questions with five responses and two questions into three responses. Total scores ranged from 0 to 5, and a score >2.5 indicating a positive attitude.

### Ethical Clearance

The ethical clearance was provided by the Research Committee of the Sherubtse College, Royal University of Bhutan [No.15 (3)-SC/Research/2020/11].

### Data Analysis

The data were downloaded from the Google Form into Microsoft Excel (Microsoft Corporation, WA, USA) for cleaning. The cleaned data were analyzed using STATA 13 (Stata Corporation, College Station, TX, USA). The descriptive data are presented in the frequencies and proportions with mean scores, SD, and range. The distribution of the KAP of the participants based on their demographics was compared using the chi-square test. The association between the knowledge, attitude, and practices was assessed using the Pearson's correlation coefficient (r).

## Results

### Sociodemography

A total of 613 students completed the survey questionnaire with a mean age of 22 years (range 18–3). Majority of the respondents were female (57.6%, 353) and third-year students 56.5% (344). More than one-third (32.6%, 199) of participants were from the Department of Arts and Humanities (32.6%) (Table 1).

**Table 1:** Sociodemographic characteristic of the study participants (*n* = 613).

**Variables**	**Mean**	**SD**	**Min**	**Max**	**Number**	**%**
Knowledge score	16.7	1.7	0	14		
Attitude score	3.7	1.0	0	5		
Practice score	5.2	1.0	0	6		
**Gender**						
Male					260	42.4
Female					353	57.6
**Age group (years)**						
<20					47	7.7
20–24					524	85.5
25+					42	6.8
**Year of study**						
First year					114	18.6
Second year					139	22.7
Third year					345	56.3
Fourth year					15	2.5
**Departments**						
Arts and humanities					200	32.6
Computer science and maths					57	9.3
Environmental and life science					139	22.7
Physical science					62	10.1
Social science					155	25.3

### Knowledge

The mean COVID-19 knowledge score was 10.7 (SD = 1.7; range: 0–14). A good knowledge score (based on 50% of the total knowledge score) was reported by 98.4% of the students. However, 87% of the students had a misconception that COVID-19 was the name of the virus rather than a disease. The majority (93.6%) of the students knew the main symptoms of COVID-19 and 95% reported that there is no effective vaccine except a supportive treatment. About 84% of the respondents correctly answered that people with COVID-19 can still transmit the disease in the absence of fever. The majority (87.5%) of the respondents knew that the virus spreads through the respiratory droplets and 88% believed that the face masks can prevent the transmission of the virus. Almost all the respondents noted that the disease can be prevented by avoidance of crowded places and large gatherings (Table 2).

**Table 2:** Knowledge on the coronavirus disease 2019 (COVID-19) among the Sherubtse College students.

**Knowledge on COVID-19**	**No (%)**	**Yes (%)**	**Don't know (%)**
K1. COVID-19 is the name of virus	79 (12.9)^*^	532 (87.1)	0 (0.0)
K2. The main clinical symptoms of COVID-19 are fever, fatigue, dry cough and body aches	15 (2.5)	573 (93.6)^*^	24 (3.9)
K3. Unlike the common cold, stuffy nose, runny nose, and sneezing are less common in persons infected with the COVID-19 virus	189 (31)	266 (43.6)^*^	155 (25.4)
K4. Currently, there is no effective cure for COVID-19 but early detection and supporting treatment can help most patients recover from the infections	6 (1.0)	582 (95.1)^*^	24 (3.9)
K5. Not all persons with COVID-2019 will develop severe illness. Only those who are elderly and have chronic illnesses are more likely to be severe cases	61 (10.0)	**512 (83.6)**	39 (6.4)
K6. Eating or touching wild animals would result in infection by the COVID-19 virus	273 (44.6)^*^	195 (31.9)	144 (23.5)
K7. Persons with COVID-19 cannot transmit the virus to others if they do not have a fever	513 (83.8)^*^	13 (2.1)	86 (14.1)
K8. The COVID-19 virus spreads via the respiratory droplets of infected individuals	23 (3.8)	533 (87.5)^*^	53 (8.7)
K9. Ordinary residents can wear face masks to prevent the infection by the COVID-19 virus	49 (8.0)	536 (87.9)^*^	25 (4.1)
K10. Children and young adults don't need to take measures to prevent the infection by the COVID-19 virus.	549 (89.7)^*^	52 (8.5)	11 (1.8)
K11. To prevent the infection by COVID-19, individuals should avoid going to crowded places	1 (0.2)	**605 (99.0)** ^*^	5 (0.8)
K12. Isolation and treatment of people who are infected with the COVID-19 virus are effective ways to reduce the spread of the virus in the community.	2 (0.3)	607 (99.2)^*^	3 (0.5)
K13. People who have contracted with someone infected with the COVID-19 virus should be immediately isolated in a proper place.	2 (0.3)	**605 (98.9)**	5 (0.8)
K14. Oral/nasopharyngeal swab is the mode of diagnosis of COVID-19	244 (40.9)	317 (53.2)^*^	35 (5.9)

* *Correct response. The bold values indicates correct answers*.

### Attitude

The mean attitude score was 3.7 (SD = 1.0; range: 0–5). A total of 531 (86.5%) students had a positive attitude while 83 (13.5%) had a poor attitude toward COVID-19. About 86% (527) of the respondents agreed or strongly agreed that COVID-19 was a very dangerous disease and over 60% (385) of them agreed or strongly agreed that COVID-19 could be successfully controlled. However, 11.3% (69) of them were unsure if COVID-19 was a dangerous disease and 30% (194) were unsure if the disease could be successfully controlled. The majority (81%) of the participants thought that Bhutan can win the battle against the virus and 97% of them were willing to help spread the preventive knowledge on COVID-19 to others. However, 42.6% reported that they were not at risk of getting the disease (Table 3).

**Table 3:** Distribution of good knowledge, attitude, and practice based on the demographic characteristics of the participants.

**Questions**	**Strongly disagree**	**Disagree**	**Neutral**	**Agree**	**Strongly agree**
A1. Do you agree that COVID-19 will finally be controlled successfully?	8 (1.3)	24 (3.9)	194 (31.8)	250 (40.9)	135 (22.1)
A2. Is COVID 19 a very dangerous disease?	3 (0.5)	12 (2.0)	69 (11.2)	192 (31.4)	335 (54.8)
			**Yes**	**No**	**Don't Know**
A3. Are you confident that we can win the battle against the COVID-19 virus?			493 (80.8)	20 (3.3)	97 (15.9)
A4. Do you think that you are at risk of getting COVID-19?			246 (40.2)	261 (42.7)	105 (17.2)

### Practice

**Figure 2:**
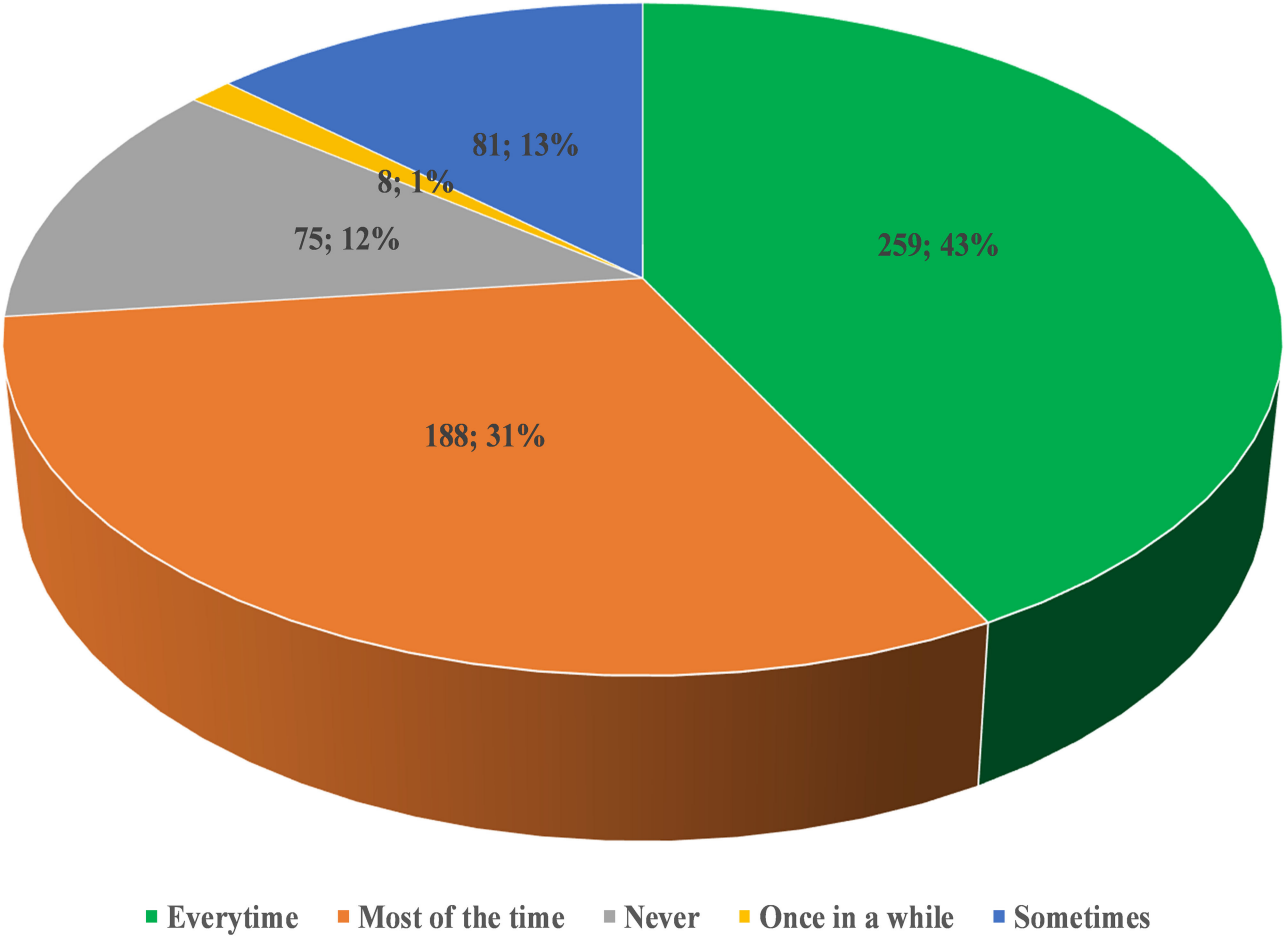
Proportion of the study participants using the “Druk Trace” app while visiting the public place (*n* = 613).

**Figure 3:**
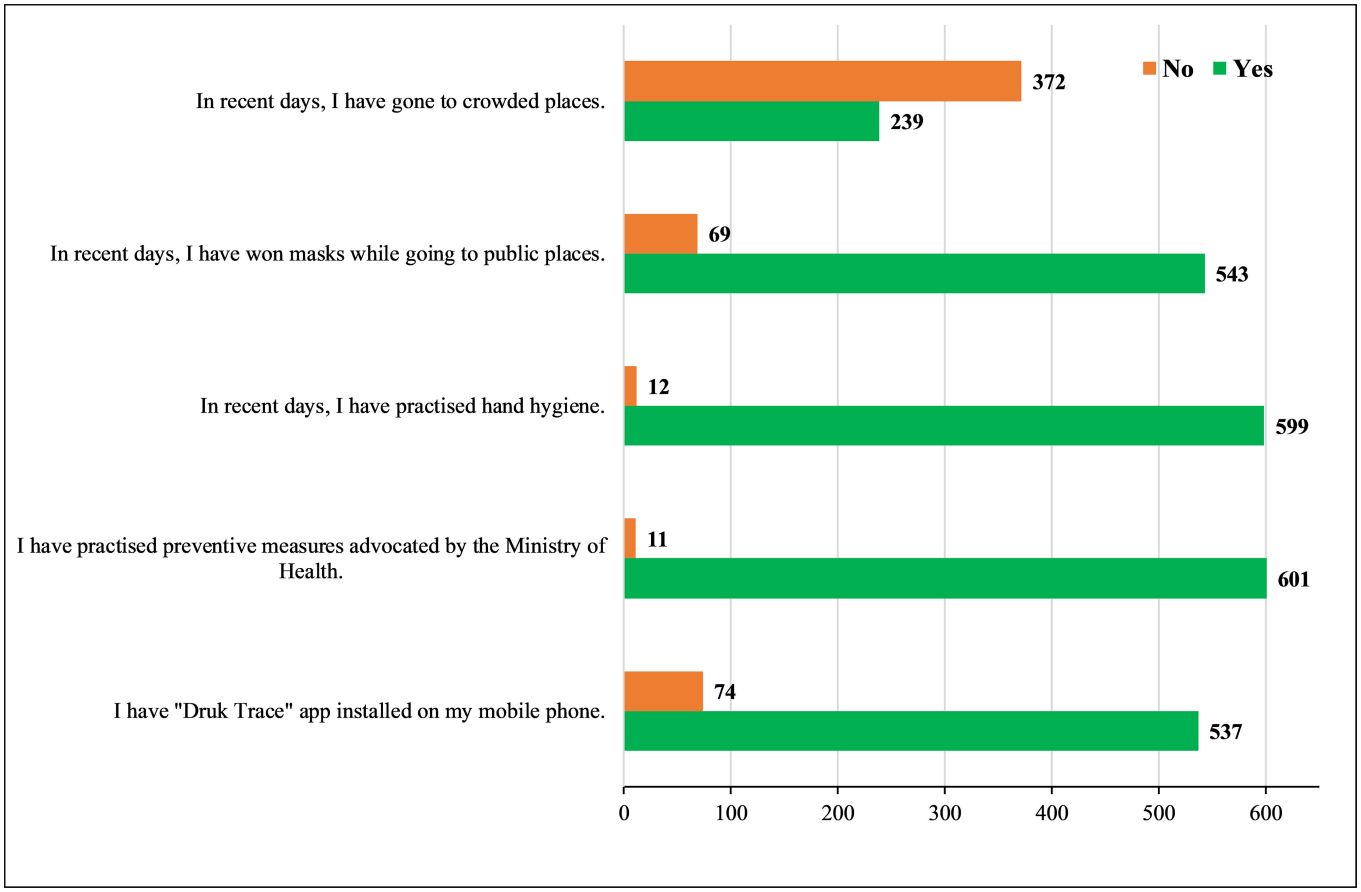
The practices of the students toward coronavirus disease 2019 (COVID-19) (*n* = 613).

The mean COVID-19 practice score was 5.2 (SD = 1.0; range: 0–6), indicating good practices (98%). More than 80% of the respondents had the “Druk Trace” App installed on their mobile phones. While 43% of the participants used it every time while visiting a public place (Figure 2). Nearly all (98%) the participants followed the preventive measures of COVID-19 advocated by the Bhutan Ministry of Health. In addition, the majority (98%) of the participants were adhering to good hand hygiene using hand sanitizer or hand washing. The face masks were worn in public places by 89% of the study participants (Figure 3).

### Association and Correlation of KAP on COVID-19

Age was associated with good knowledge (*p* = 0.023). While the year of study was associated with a positive attitude (*p* = 0.026) and good practice (*p* = 0.048; Table 4). The correlation analysis indicated the presence of a positive but weak correlation between good knowledge and practice (*r* = 0.1, *p* = 0.0126), having a positive attitude led to practicing appropriately most of the time (*r* = 0.1866, *p* < 0.001; Table 5).

**Table 4:** Distribution of good knowledge, attitude, and practice based on the demographic characteristics of the participants.

**Variables**	**Good knowledge**		**Positive attitude**		**Good practices**	
	***N* (%)**	***P***	***N* (%)**	***P***	***N* (%)**	***P***
**Age (years)**						
<20	44 (93.6)	0.023^*^	39 (83.0)	0.580	45 (95.7)	0.475
20–24	518 (98.9)		457 (87.2)		515 (98.3)	
25+	41 (97.6)		35 (83.3)		41 (97.6)	
**Sex**						
Male	256 (98.5)	0.876	227 (87.3)	0.669	252 (96.9)	0.086
Female	347 (98.3)		304 (86.1)		349 (98.9)	
**Year**						
First year	111 (97.4)	0.642	90 (79)	0.026^*^	112 (98.3)	0.048^*^
Second year	136 (97.8)		118 (84.9)		130 (95.6)	
Third year	341 (98.8)		310 (89.7)		341 (99.1)	
Fourth year	15 (100.0)		13 (86.7)		14 (93.3)	
**Department**						
Arts & Humanities	197 (98.5)	0.806	168 (84)	0.103	192 (96.0)	0.130
CSM^**^	56 (98.3)		53 (93)		57 (100.0)	
ELS^†^	138 (99.3)		123 (88.5)		137 (98.6)	
Physical science	61 (98.4)		58 (93.6)		61 (98.4)	61 (98.4)
Social science	151 (97.4)		129 (83.2)		154 (99.4)	

* *Significant at p < 0.05;*

** *Computer science and mathematics;*

† *Environmental and life science. The bold values indicate significant values (p-values < 0.05)*.

**Table 5:** Correlation between knowledge, attitude, and practices.

**Scales**		**Knowledge**		**Attitude**		**Practice**	
		**Good**	**Poor**	**Good**	**Poor**	**Good**	**Poor**
Knowledge	Good			525 (87.1)	78 (12.9)	591 (98.0)	12 (2.0)
	Poor			6 (60.0)	4 (40.0)	10 (100.0)	0 (0.0)
Attitude	Good	*r* = −0.0182				526 (99.1)	5 (0.94)
	Poor	*p* = 0.653				75 (91.5)	7 (8.5)
Practice	Good	*r* = 0.1		*r* = 0.1866			
	Poor	***p*** = 0.0126^*^		***p*** < 0.001^*^			

* *p < 0.05. The bold values indicate significant values (p-values < 0.05)*.

## Discussion

In the present study, we evaluated KAP toward COVID-19 among the students of Sherubtse College in Bhutan. This study showed 98% had a good level of knowledge, 87% had a positive attitude, and 98% reported good practice on COVID-19. There was a weak but positive correlation between the good knowledge and practice, and a positive attitude and practice.

The overall COVID-19 knowledge among the students in this study was good. Even though the study population was different, similar findings with good knowledge were reported in China (18, 22), Cameroon (23), Malaysia (24), Iran (25), and Egypt (26). While a poor level of knowledge toward COVID-19 was reported in Ethiopia (27) and Bangladesh (28). The high knowledge score in this study is not surprising due to the characteristics of the study sample being a college student. In addition, this study was conducted during massive public health education and awareness campaign by the government of Bhutan through all the available mass media, such as newspapers, television, radio, and various social media (Facebook). It would be important to evaluate the level of knowledge in the general population in Bhutan.

The study participants showed a positive and optimistic attitude toward COVID-19. The positive attitudes can be attributed to the response of the Bhutan government to the pandemic. After the first case of COVID-19 in Bhutan on March 5, 2020. Bhutan initiated 21 days mandatory quarantine for the returning travelers from a third country, 7 days quarantine for people moving from the border towns with India to interior districts, and snap lockdown of local areas when a community transmission is reported (3, 4). In addition, mandatory use of face mask in public places and restrictions to the mass gathering.

The overall practice score on the prevention of the COVID-19 in this study was much better than elsewhere (27). The majority of the respondents stated that they used a face mask in the public places and other mass gatherings. Similar findings were reported from China and Ethiopia (18, 27). The use of a face mask is known to prevent transmission of the virus (29). One of the reasons for the consistent use of the face mask by the students could be a result of a mandatory use imposed by the government. In addition, the compliance of the face mask is strictly monitored by police and other volunteers. The findings of this study were much better than among the university students in Bangladesh where only half of the students reported wearing face masks (21). The difference could be due to the study period because the Bangladeshi study was conducted in the early phase of the pandemic. Furthermore, monitoring and implementation in the populated countries, such as Bangladesh might be difficult unlike in Bhutan.

There was a weak but positive correlation between knowledge and practice, and attitude and practice. A similar correlation between positive knowledge and practice (28, 30, 31), and attitude and practice (28, 30) were reported in other studies. A systematic review on KAP among the general population showed studies in Asia had comparatively good KAP compared with the Americans (32). This could be the reason that the preventive actions taken as a result of good knowledge led to a lesser number of cases in Asia compared with the United States. It has been shown that good knowledge can enhance the positive preventive behaviors through early identification of disease and better health-seeking behavior (33). In addition, knowledge affects the behavior of individual and a higher knowledge level reinforces healthier behaviors (34), such as social distancing, avoiding mass gathering, and shaking hands (30). Such positive practices are important for breaking the transmission cycle of COVID-19 in the community.

The policy implication of this study is that the students had a high COVID-19 knowledge with a positive attitude and adhering to the recommended preventive measures. Therefore, reopening schools and colleges can be considered with minimal risk of COVID-19 transmission. Second, the current health education toward COVID-19 seems to be effective in this group of audience. Third, the students in Bhutan can be an important source of information for their immediate families because most of the older generation are illiterate.

### Conclusion

This is the first study to investigate KAP toward COVID-19 among the students in Bhutan. The students had good KAP scores and followed COVID-19 protocols advocated by the government. Good knowledge and a positive attitude were translated into good practice. Therefore, the education campaign of Bhutan government seems to be effective in the students.

### Recommendations

Although the overall KAP score was good in this study, we recommend a similar study with a larger sample size, such as students from other schools. Further, a community-based KAP study in the general public needs to be undertaken to evaluate the effectiveness of the education program initiated by the Bhutan government in the general population. The study findings will be useful to inform the policymakers and healthcare professionals, on the future public health interventions, awareness-raising, policies, and health education programs.

### Limitations

The findings from this study need to be interpreted with the consideration of some limitations. First, causal inferences cannot be established due to a cross-sectional study design. Second, other socio-demographic characteristics could not be collected due to the similar characteristics of the student. Hence, future studies should use methods that will explore both the linear and non-linear relationships between socio-demographic characteristics and COVID-19. Third, due to the self-reporting of the survey questionnaire, there is a risk of reporting bias. This can be addressed in the future through a face-to-face interview. Fourth, some answers may not have been given honestly due to social desirability. In addition, this study was conducted among the college students, who are educated, and therefore, the findings cannot be generalized.

## Data Availability Statement

The raw data supporting the conclusions of this article will be made available by the authors, without undue reservation.

## Ethics Statement

The studies involving human participants were reviewed and approved by College Research Committee, Sherubtse College, Royal University of Bhutan. Written informed consent for participation was not required for this study in accordance with the national legislation and the institutional requirements.

## Author Contributions

TD conceived, undertook the study, analysis, and drafted the manuscript. KWangmo helped in the statistical analysis and interpretation of results and revision of the manuscript. KWangdi assisted in data collection, analysis, and revision of the manuscript. Y, TW, and T assisted in the revision of the manuscript. All the authors read and approved the final draft.

## Conflict of Interest

The authors declare that the research was conducted in the absence of any commercial or financial relationships that could be construed as a potential conflict of interest.

## Publisher's Note

All claims expressed in this article are solely those of the authors and do not necessarily represent those of their affiliated organizations, or those of the publisher, the editors and the reviewers. Any product that may be evaluated in this article, or claim that may be made by its manufacturer, is not guaranteed or endorsed by the publisher.
